# Notes and News

**Published:** 1896-02-08

**Authors:** 


### Notes and News.

(Collected and Communicated.)

A fellowship in anatomy in honour of the late Dr. Leidy has been established at the University of Pennsylvannia.

Mr. Henry Tate has given £5,000 to the Liverpool Infirmary. The building is a very fine one, but costly to maintain, and therefore this addition to its resources will be very welcome.

His Royal Highness the Duke of York has fixed May 21st as the date upon which he will preside at a festival dinner in aid of the Royal Albert Orphan Asylum (Bagshot) for necessitous boys and girls.

At the last general meeting of the Edinburgh Infirmary the use of whiskey for patients instead of brandy was advocated by a layman, who, however, admitted that the decision must rest with the medical staff.

The method of photography reputed to be Professor Rœntgen's discovery has been known and utilised by an Oxford professor for some time. Unfortunately for his fame, he never applied the method to the human body, devoting its use in research in mineral substances.

Through the will of the late Mr. David James, a number of charities have received bequests. The Brompton Hospital for Consumption has received £1,000, Charing Cross Hospital £1,000, Central London Throat and Ear Hospital and the Hospital for Sick Children £500, Royal Hospital for Incurables £500, and Guy's Hospital £1,000.

A cottage hospital has been opened at Ormskirk by the Countess of Derby. The institution was much wanted in the district. The hospital will consist of three blocks, connected by a corridor, and affording accommodation for eight patients. The central block only has been provided at present, which provides no accommodation for the administration, consequently temporary arrangements have had to be made.

C. Gordon Brodie, F.R.C.S.Eng., has been appointed assistant surgeon to the City Orthopædic Hospital.

An examination of candidates for fifteen commissions in the medical department of the navy is to be held in May. Full particulars will be found in our advertisement columns.

The Attorney-General has consented to preside at a festival dinner in aid of funds for the Royal National Hospital for Consumption and Diseases of the Chest, at the Whitehall Rooms, Hotel Métropole, on Wednesday, April 29th.

H.R.H. the Princess of Wales has been graciously pleased to become patroness of the concert to be given, by kind permission of the Duchess of Sutherland, at Stafford House on Thursday, June 11th, in aid of the building fund of the Free Home for the Dying, Clapham. All communications should be addressed to the Ladies' Committee, at the office of the Hon. Secretary, Captain Portlock-Dadson, 281, Strand.

A monument to Pasteur is to be erected in his native town of Dôle. A subscription list has been opened, to which contributions from all nations are invited. M. Felix Faure presides over the committee. M. Marius Duché, president of the French Chamber of Commerce in London, will be willing to receive subscriptions in England. The Medical Society of Berlin has sent a subscription of 1,000 frs. to Dôle.

In July last we gave an illustrated description of a caravan hospital established in Haddingtonshire to be used for infectious diseases, and moved about from district to district as required. The scheme was a new one, and from the point of view of its cheapness had sufficient to recommend it for us to follow its progress with interest. Experience has proved that the system is far from satisfactory. It appears that the hospital, when situated near running water, has been the means of spreading disease, and in one case both the attendants on a patient suffering from enteric fever caught and succumbed to the malady. There appears to be sufficient evidence forthcoming to show that the system, as carried out at present, is a failure, and unless more sanitary conditions can be obtained, we hope that the experiment will not be pursued any further.

In the earlier days of the Madagascar Expedition a general impression was prevalent that the medical department of the French army was admirably equipped and organised. We are now informed that it is an object lesson to the contrary. The mortality amongst the troops reached upwards of 4,000, and the inference drawn is, that an absence of proper hygienic precautions and medical aid had much to do with the appalling loss of life. It is stated that the advice of the medical staff was frequently set aside, that medical materials were most insufficient, and over-crowding in the hospitals was permitted. All these factors, added to the malarious nature of the country, the youth of the men, and the heavy baggage and accoutrements with which they were burdened, united with overwhelming force against the health of the army, and resulted in terrible loss of life. The French Army Medical Service is said to be lacking in control, the absence of authority on the part of its officers being disastrous when action is necessary. It is to be hoped, for the sake of the troops, that expert advice may have a better chance when next the French nation embarks in warfare.

